# Who's minding the shop? The role of Canadian research ethics boards in the creation and uses of registries and biobanks

**DOI:** 10.1186/1472-6939-9-17

**Published:** 2008-11-14

**Authors:** Elaine Gibson, Kevin Brazil, Michael D Coughlin, Claudia Emerson, Francois Fournier, Lisa Schwartz, Karen V Szala-Meneok, Karen M Weisbaum, Donald J Willison

**Affiliations:** 1 Health Law Institute, Dalhousie University, Halifax, Canada; 2 Department of Clinical Epidemiology & Biostatistics, McMaster University, Hamilton, Canada; 3 Centre for Evaluation of Medicines, St. Joseph's Health Care, Hamilton, Canada; 4 Department of Psychiatry and Behavioural Neurosciences, McMaster University, Hamilton, Canada; 5 Program on Ethics and Commercialization, McLaughlin-Rotman Centre for Global Health, University Health Network and University of Toronto, Toronto, Canada; 6 Centre de bioéthique, Institut de Recherches Cliniques de Montréal, Montreal, Canada; 7 School of Rehabilitation Sciences, McMaster University, Hamilton, Canada; 8 St. Joseph's Health System Research Network, Hamilton, Canada; 9 Division of Palliative Care, Family Medicine, McMaster University, Hamilton, Canada; 10 Department of Family Medicine, Queen's University, Kingston, Canada

## Abstract

**Background:**

The amount of research utilizing health information has increased dramatically over the last ten years. Many institutions have extensive biobank holdings collected over a number of years for clinical and teaching purposes, but are uncertain as to the proper circumstances in which to permit research uses of these samples. Research Ethics Boards (REBs) in Canada and elsewhere in the world are grappling with these issues, but lack clear guidance regarding their role in the creation of and access to registries and biobanks.

**Methods:**

Chairs of 34 REBS and/or REB Administrators affiliated with Faculties of Medicine in Canadian universities were interviewed. Interviews consisted of structured questions dealing with diabetes-related scenarios, with open-ended responses and probing for rationales. The two scenarios involved the development of a diabetes registry using clinical encounter data across several physicians' practices, and the addition of biological samples to the registry to create a biobank.

**Results:**

There was a wide range of responses given for the questions raised in the scenarios, indicating a lack of clarity about the role of REBs in registries and biobanks. With respect to the creation of a registry, a minority of sites felt that consent was not required for the information to be entered into the registry. Whether patient consent was required for information to be entered into the registry and the duration for which the consent would be operative differed across sites. With respect to the creation of a biobank linked to the registry, a majority of sites viewed biobank information as qualitatively different from other types of personal health information. All respondents agreed that patient consent was needed for blood samples to be placed in the biobank but the duration of consent again varied.

**Conclusion:**

Participants were more attuned to issues surrounding biobanks as compared to registries and demonstrated a higher level of concern regarding biobanks. As registries and biobanks expand, there is a need for critical analysis of suitable roles for REBs and subsequent guidance on these topics. The authors conclude by recommending REB participation in the creation of registries and biobanks and the eventual drafting of comprehensive legislation.

## Background

The amount of research utilizing health information has increased dramatically over the last ten years. Single, time-limited studies with tightly-defined research questions are giving way to programs of research that rely upon the systematic prospective collection of data in registries and biobanks for subsequent use in multiple projects to answer as yet unknown research questions. Many institutions have extensive biobank holdings collected over a number of years for clinical and teaching purposes, but are uncertain as to the proper circumstances in which to permit research uses of these samples. Research Ethics Boards (REBs) in Canada and elsewhere in the world are grappling with these issues, but have not received clear guidance regarding their role in the creation of and access to registries and biobanks. Historically, REBs have played an active role regarding specific project-by-project requests exclusively, thus not engaging in some of the larger issues concerning the creation and research uses of registries and biobanks. Indeed, some may not even be aware of the extent of registry and biobank holdings within their institutions. REBs we interviewed expressed concern and confusion both as to the handling of specific projects emanating from registries and biobanks, and to the broader issues surrounding them.

In this paper we outline variation in the responses of a wide sampling of REBs across Canada to a series of questions regarding the creation and use of registry and biobank information for health research purposes, and we note the heightened tension surrounding biobanks. We then discuss the implications of our findings for the development of policy and legislation.

## Methods

### Design & Sample

We approached the Chairs of 34 REBs affiliated with Faculties of Medicine in Canadian universities and requested interviews with them and/or with their REB Administrators. They were also invited to include other REB members in the interview. The interview was to be 90-minutes face-to-face. Ethics approval was obtained from Research Ethics Boards at the universities of McMaster, Dalhousie, and Montreal and at St. Joseph Healthcare, Hamilton, Ontario.

### Procedure

Interviews consisted of structured questions dealing with diabetes-related scenarios, with open-ended responses and probing for rationales. The two scenarios discussed in this paper involved the development of a diabetes registry using clinical encounter data across several physicians' practices, and the addition of biological samples to the registry to create a biobank [1]. All interviews but one were audio-recorded.

### Scenarios

The registry scenario involved the construction of a multi-centre multi-jurisdictional diabetes registry to serve as an ongoing resource for conducting epidemiologic and process-outcome studies. No specific research questions were identified. Instead, this was intended to provide a general research platform for future epidemiologic studies. The plan was to collect data through physician practices. At a regular patient visit, the physician would complete a duplicate encounter form. One copy would go in the patient's file; the second would be supplied to the research associate at the principal investigator's office, who was then to remove any direct identifiers and forward the data to the central registry. This registry was intended to be updated during routine patient care visits, and was to continue indefinitely. The research associate would hold the identification key in order to link newly received information with that already received for the particular patient.

[See additional file 1]

In the biobank scenario, blood samples were to be taken from patients during, but in addition to, routine patient care. The samples were to be retained indefinitely. The information garnered from the blood samples would be linked with the diabetes registry using a common study ID. The combined biobank and registry were intended to serve as an ongoing resource for studying biological markers of diabetes and conducting pedigree studies.

[See additional file 2]

### Main Outcome Measures

Major questions asked were as follows:

• In terms of the *creation of registries*, we asked whether or not patient consent is required for inclusion in a registry, and the rationale. We also queried the duration of consent – that is, whether it should last for the duration of the registry or if periodic renewal would be required – and the reasoning behind their views on duration.

• As to the *operation of registries*, we inquired into the need (or lack thereof) for ongoing monitoring of the registry by the REB, and the types of information that would need to be reported. We also probed whether the REB would review the individual research projects utilizing the registry, and the factors that contributed to this decision.

• We asked whether the REB viewed *biobanks *as qualitatively different from registries, and the reasons behind their views. Further, we queried the need (or not) for consent and, if needed, its duration.

• We also inquired into any additional reporting requirements surrounding *biobanks*.

### Embedded Issues

A number of issues were built into the scenario. These included the implications of requesting REB review without a specific set of research questions attached to the creation of the registry/biobank, but rather only a general research agenda. In the case of the registry, the data were to be sent offsite to the principal investigator's office for coding and removal of identifiers. For the biobank, a common study ID would be used for both the biological samples and the clinical data in order to facilitate linkage.

### Analysis

Interviews were transcribed, checked for accuracy against the original audio recordings, and forwarded to interviewees to review for accuracy and for clarification where the initial response may have been unclear. Transcript review moved through several iterations that can be summarized into two stages. In stage 1, all co-investigators reviewed the first 11 interviews and, based on these, identified themes and sub-themes to pursue in the analysis and response categories. In stage 2, the interviewers and a graduate student reviewed all transcripts (including those that had been reviewed in stage 1), coded responses according to the themes identified, and summarized respondents' rationales. In some cases, additional themes emerged or additional nuances were identified for the original themes. When responses were difficult to categorize, the P.I. independently coded these sections. Answers and rationales were then discussed as a group to reach consensus. In a few remaining instances, answers were not classifiable due to a lack of clarity; this is noted where applicable in the results section.

To support the interpretations drawn by the researchers, short examples or typical statements have been included in the text. Quotations are presented in italics. Minimal editing has been done to preserve authenticity while ensuring readability.

## Results

Thirty 90-minute face-to-face interviews were conducted with Chairs and/or Administrators (response rate 88%). In some cases, one or more other REB members also attended, to a maximum of seven in attendance. The median number of attendees was two.

### Registry

Of the thirty sites, one refused to entertain the scenario regarding the creation of the registry, indicating that its creation was not connected with any specific research question and therefore falls outside its mandate. Their concern was a blurring of the concept of creation of research infrastructure with that of review of research protocols, and that approving the infrastructure would open the door to unapproved data uses by the researcher. The remainder of questions in this section were skipped for this site. The other twenty-nine sites responded.

In response to the question as to whether patient consent is required for inclusion of her/his data in the registry, twenty-three of twenty-nine sites answered affirmatively. Reasons included the planned collection of identifiable data; the intention to utilize the data for research in future; the fact that identifiable data would be going offsite; and the plan to collect data prospectively, meaning that there would be ongoing contact with the patients and therefore seeking consent would not be onerous. Sixteen of these sites indicated they would not be sympathetic to an argument by the researcher that seeking individual consent is impracticable.

Six sites indicated that consent would not be required for creation of the registry. Three of these did not consider this to be research; one indicated "this sounds more like ongoing chart review", and another that "it's an exploratory study on a large volume of data." Two of the sites would not require consent because the data would be stripped of direct identifiers prior to its entry into the registry. Two sites not requiring consent would place conditions on the creator of the database – i.e., either an information letter to patients or notification with opt-out.

Of the twenty-three sites that would require consent, there was a high degree of variation as to limits on its duration. Twelve agreed, but for differing reasons, that the patient's consent would run for the duration of the registry in the absence of significant change. Of these, two sites saw no reason for periodic renewal of consent; five indicated providing an option to withdraw would obviate the need to require consent renewal; and three sites were motivated by the fact that they would require consent for specific research studies utilizing the registry. Another reason given was that the registry would lose scientific validity over time if periodic consent were required. Five sites would require periodic renewal, with the periods ranging from every subsequent patient visit to once every five years. This was viewed as feasible given that the patients are to be followed in the course of routine clinical care. Four sites were undecided, indicating that the duration of consent would be decided on a case-by-case basis. One site's answer was indecipherable and we were unable to get clarification on follow-up.

Twenty-four of twenty-nine sites would require periodic reporting to the REB by the registry custodians. Along with standard information for progress reports, the content of such reports would include registry-specific information such as how the registry is being managed, who has access, the evolution of the population (i.e., enrolments and withdrawals), and the source(s) of funding of the registry. In the case of the five remaining sites, one was undecided, in one case the answer was unclear, in one case the question was skipped, one indicated it would only require reporting in case of amendments to the registry, and the final site would not require periodic reporting due to a lack of resources for follow-up.

The twenty-nine sites were also asked whether specific research projects utilizing the registry would require REB review. Twenty answered in the affirmative, although three of these twenty indicated that such review would likely be expedited (i.e., not reviewed by the full REB). One site said 'no', while six provided responses conditional on the circumstances; for three of the six, review would not be required if the data was de-identified; for two, it would depend on whether there were substantial changes to the protocol consented to upon establishment of the registry; and for one, review would only be required if dramatically different uses were to be made of the data (e.g., linkage to blood samples). Two sites were undecided.

### Registry Combined with Biological Samples

One REB viewed the creation of a biobank with linkage to the registry information as outside the scope of REB scrutiny (the same REB that had indicated that registry creation was outside its scope). Of the remaining twenty-nine sites, twenty-three viewed the biobank information as qualitatively different from other types of personal health information, while six indicated the difference was at most a question of degree. One site stated that there is no difference; all information requires sensitive handling, whether or not it has genetic markers. Those that stated the difference is one solely of degree generally regarded information from the biological sample as being more sensitive, replicable, commercializable, and predictive. Of the sites that viewed it as qualitatively different, reasons given were its intra-familial and inter-generational nature; the implications for insurability and employability; the potential uses in deciding on paternity; and its regional or group implications, including discrimination on the basis of race.

The twenty-nine sites were unanimous with regard to the need for patient consent for blood samples to be placed in the biobank. All six of the sites that had not required consent for participation in the registry would now do so.

In terms of duration of consent for the biobank samples, six stated that its duration was time-limited, eleven indicated there would be no time limit for retention, five were undecided, in three cases the answer was unclear, and in four cases the question was skipped. Note that two of the sites that would require periodic re-consent for the registry alone would not require it when the biological samples were combined with the registry. These findings demonstrate indeterminacy on the part of REBs, as revealed by one site's statement: "We don't have to provide answers to all these questions. They're not all answerable."

Of the six sites who said consent would be time-limited, one stated that "blood is different" and another that its potential uses are endless, unlike registries. For some of these sites, samples or linkages would be destroyed after a set time period, ranging from five to twenty-five years. Those indicating no time limit to consent sometimes included one or more qualifiers, such as a withdrawal option, and notification should there be significant changes to the biobank. Some additional requirements identified were: full REB review in the first year of operation; bio-collection treated as a separate protocol; periodic report on the activities and outcomes of research using the biobank; and scrutiny of physical security measures. As with the diabetes registry, one site would not require periodic reporting due to lack of REB resources.

### Study Limitations

Interviewees had been told that the interviews would take at maximum ninety minutes. Since questions were open-ended, it was necessary at times that the interviewer skip some questions in order to complete in the promised time frame. This led to some incompleteness in results.

Note that this study was constructed around hypothetical situations. An REB faced with a real-life application for approval would have the opportunity to request further details and to deliberate at length. Further, the outcomes measure was what sites *said *they would do, based on these hypothetical facts. Not all of the sites had handled all of the types of requests included in our scenarios. Thus, some of the answers may have reflected their understanding of current guidelines, rather than reflecting past practice. In addition, responses may have been shaped in accordance with what the interviewee expected the interviewer wished to hear.

## Discussion and Conclusion

We found that participants were more attuned to issues surrounding biobanks as compared to registries, despite similarities regarding their creation and long-term research potential. This is not surprising given that the Tri-Council Policy Statement (TCPS), a statement agreed to by the major federal research funding agencies in Canada which aims to ensure the ethical conduct of research, is silent as to registries. Also, there is a dearth of literature, both in Canada and internationally, concerning the role of REBs vis-à-vis registries. There is also a significant degree of variation in how the sites in our survey indicated they would handle research proposals for creation and use of these entities. For example, six of the twenty-nine sites entertaining the scenario would not require patient consent for the entry of personal information into a registry, whereas all twenty-nine would require consent for entry of blood samples into a biobank. At least two factors are at play in creating the consensus as to biobanking. First, participants saw the scope of potential research activities to be much more broad for biobanks in comparison to registry information. Indeed, the limits for biobanks were identified as unknowable. Second, the TCPS does contain guidance as to human tissue, including the explicit requirement of informed consent to its collection and use [2].

Accompanying this greater familiarity is a dramatically higher level of concern on the part of sites regarding biobanks. One referred to biobank information as a 'gray box' in that its potential future uses are at present unknowable. Others referred to such information as providing a "total picture of the person" or "a window into one's soul", and that "the sum is greater than the parts". These vivid and dramatic descriptors are indicative of trepidation on the part of participants regarding genetic information. There is a significant degree of ambivalence in the literature on biobanks as to whether or not genetic information is inherently different from other types of health information. Some argue that all personal health information is potentially sensitive [3]. Others lean to "genetic exceptionalism"[4,5] despite the fact that other types of information may also implicate family or community as well as the individual, and may be highly sensitive (e.g. HIV status or psychiatric record). The majority of sites in our study (23/29) viewed genetic information as qualitatively different, thus weighing in on the 'exceptionalism' side of the debate.

Given this acutely higher level of concern regarding biobanks, it is surprising that an equal number of sites would not require periodic renewal of consent for registries and for biobanks. Specifically, sixteen sites would permit the entry of information into a registry to run indefinitely or were undecided, and sixteen sites would either permit consent to banking of a blood sample to continue indefinitely into the future or were undecided. One of the sites that would not require periodic renewal of consent for the biobank in contrast to the registry provided this explanation: "No, because once the sample is given, it's for life, you don't go a second time...up to now it's once and for all." These findings give rise to serious concern about consent practices regarding biobanks, especially since the samples are often retained long-term. After describing the general standards for informed consent for research involving human subjects in the U.S., Natalie Ram notes that " [a]gencies and courts have been hesitant to impose similar consent requirements on researchers obtaining human tissue for use in research, and human tissue research has therefore become a particularly thorny problem for traditional formulations of informed consent."[6]

One of the fascinating differences between sites with regard to their concerns or lack thereof with identifiability of registry data revolves around at what point in the process they were focussing on. Sites with concerns looked at an earlier period of time than entry into the registry – i.e., the fact that identifiable data were to go offsite to the principal investigator's office prior to being de-identified. One site indicated they would simply not allow release of personal information out-of-house, as had been proposed. The sites that were not concerned indicated that the data was de-identified upon entry into the registry. At least one REB member expressed trust that researchers would safeguard the personal information and not attempt to re-identify individuals.

Expansion continues apace for registries and biobanks. This results in a need for critical analysis of suitable roles for REBs and subsequent guidance on these topics. A first step is to establish a dialogue on these issues, especially regarding registries; it is hoped that this project facilitates such discussion. Registries are of burgeoning importance in response to demands for evidence-based decision-making and the growth in numbers of epidemiological studies. They give rise to a number of privacy and consent issues that outstrip current guidance and yet will need to be dealt with by REBs.

A second step will be the provision of urgently needed guidance regarding appropriate uses of information in biobanks and registries. One site referred to the rapidly changing context of genetics, and indicated that "...we're still disoriented." Sections of the TCPS on biobanking have not been updated since 1998 despite significant changes in practice combined with a huge expansion in their importance and significance. The Canadian Institutes of Health Research has developed a voluntary Best Practices code for the handling of personal information in health research [7]. While registries are addressed in the document, recommendations are currently very broad; more specific guidance as to both registries and biobanks would be in order for future editions. Further, we call on the Interagency Panel on Research Ethics to undertake a review and redrafting of parts of the TCPS of direct relevance to registries and biobanks. Accompanying this should be an education programme covering these topics aimed at researchers, REB members, and privacy commissioners.

Third, we urge that REBs adopt an active role in guiding the creation of registries and biobanks. This holistic approach responds to the development of multi-project research platforms as opposed to simply individual projects. Several sites were concerned about jurisdiction and lack of specificity in being asked to review infrastructure; one site indicated that "we shouldn't be collecting data until we know what the future use might be...this is just a little too wide open, it's a fishing expedition." However, it is our position that since registries and biobanks are indeed being created, it makes sense that any obvious potential problems be addressed up-front, prior to the infrastructure being developed. This will result in greater efficiency and less work later for both researchers and REBs, and the avoidance of future problems. The concern about a 'fishing expedition' can be allayed by the fact that the individual research projects relying on the platform should still be subjected to REB review.

Fourth, the development of one or more specialized REBs with expertise in the area of registries and biobanks is well worth considering. Models that could be adapted exist in the form of committees that specialize in screening access to databases and in the governance of biobanks [8].

And finally, in the longer term, we suggest the development of governing legislation. This would provide a superior form of guidance and control, given the sensitivity of personal health information generally and of genetic information in particular. This is particularly pertinent if use and/or disclosure of personal health information, including genetic material, without consent is under consideration. Caulfield et al. suggest that an authorization model for genetic databases may be superior to the present consent regime, but that legislation would be needed prior to adopting such a model [9,10].

The development of legislation would not be free of complexities. For example, health and information are both primarily within provincial jurisdiction. All provinces have legislation governing information in the public sector, and most now also have legislation that covers aspects of information-handling in the private sector. There is also federal private sector legislation [11]. There is a lack of consistency as to the impact of these various statutes on the conduct of research, and often a lack of clarity. Thus, drafters of legislation would need to take into account these issues and contingencies. However, the difficulties are not insurmountable, and there is an obvious sense of need. To paraphrase one of the sites in our study:

Some of the decisions should be taken by authorities above local REBs. Rules should be clarified so that each local REB does not have to take decisions. Such fundamental decisions [should] not rest upon the shoulders of local REBs. The consequences of certain decisions can impact on people or populations, which makes even more [persuasive] the case for the need for a regulatory framework on banks.

## Competing interests

The authors declare that they have no competing interests.

## Authors' contributions

EG was primary author of this paper. DW participated substantially in its revisions and was the primary investigator for the larger project of which the study of registries and biobanks formed a part. Other authors participated in the project and provided feedback on drafts.

## Pre-publication history

The pre-publication history for this paper can be accessed here:



## Supplementary Material

Additional file 1 **Creating a Diabetes Registry.** Scenario for Registry. Click here for file

Additional file 2 **Prospective Collection of Biological Samples for Diabetes Biomarkers and Pedigree Studies.** Scenario for Biobank. Click here for file
