# Supplementary material for: Who's minding the shop? The role of Canadian research ethics boards in the creation and uses of registries and biobanks
Source: BMC Med Ethics. 2008 Nov 14;9:17. doi: 10.1186/1472-6939-9-17 (PMC2636819; doi:10.1186/1472-6939-9-17)
Supplement: Additional file 1 — Creating a Diabetes Registry. Scenario for Registry. [file 1472-6939-9-17-S1.doc]

## 1. Creating a Diabetes Registry

### Research Question

- In this scenario, the researchers are part of a broad multi-centred network of researchers interested in developing a clinical diabetes registry with a view to conducting some broad epidemiologic studies and process-outcome studies in a "real-world" setting.
- Some general research questions have been identified, but the intention is to have a readily identified cohort of patients in which different research questions can be asked. However, there is no single research question being asked.

### Summary of Research Methods

A. Patients will be accrued and data collected through family physicians' practices.

B. Physicians will use a standardized paper-based form for recording any encounter with their diabetic patients. There will be an initial duplicate encounter form completed that includes question about

- Sex
- Date of birth
- Family/personal history
- Age at onset of symptoms
- Height
- Weight
- Dietary habits

In addition, ongoing tests and therapies will be recorded on a duplicate form.

C. All forms will have a copy where the original becomes a part of the medical record and the copy gets forwarded to the office of the local principal investigator, where a research assistant enters the information into a database. The copy includes patient name, physician name and health card number to identify the individual when updating the research database. The research assistant maintains a separate file with these identifying data elements and a study ID.

D. Every two weeks, the research assistant submits new data on CD by courier to a central site outside the province, where the data from across the country are housed. The data submitted to the central repository have all direct identifiers removed. The file that links study number with identifying information remains at the site of the local principal investigator. The researchers intend to follow each patient and to maintain the registry indefinitely.
