# Supplementary material for: Who's minding the shop? The role of Canadian research ethics boards in the creation and uses of registries and biobanks
Source: BMC Med Ethics. 2008 Nov 14;9:17. doi: 10.1186/1472-6939-9-17 (PMC2636819; doi:10.1186/1472-6939-9-17)
Supplement: Additional file 2 — Prospective Collection of Biological Samples for Diabetes Biomarkers and Pedigree Studies. Scenario for Biobank. [file 1472-6939-9-17-S2.doc]

## 2. Prospective Collection of Biological Samples for Diabetes Biomarkers and Pedigree Studies

### Research Question

- The diabetes registry in Scenario 3 has been successfully running for a couple of years. Let's go back, though, to the assumption that there is no contacting of patients outside their usual care.
- The investigators now wish to embark on a new phase—the prospective collection and banking of blood samples from these patients for future studies of biological markers of diabetes and for pedigree studies related to these biological markers.

### Summary of research methods

A. Samples will be collected over time as patients in the registry come for their usual and customary care. There will be no special wave of solicitation among those already in the registry to obtain blood samples for banking.

B. For the purposes envisioned, researchers will need to maintain links between the biological samples and the clinical information available in the registry.

C. As the central database has no directly identifying information, the investigators propose that the clinical information simply be directly linked with the biological sample through the common study ID

D. As with the clinical data being collected, researchers plan to keep these samples indefinitely, as the potential future uses are unknown.

E. Also, researchers want to contact "immediate" family members (parents, siblings, children, aunts/uncles, and first cousins) to conduct pedigree studies. However, they are unsure as to how best to approach these family members and seek your advice.
